# Multi-scale and cross-dimensional TMS mapping: A proof of principle in patients with Parkinson’s disease and deep brain stimulation

**DOI:** 10.3389/fnins.2023.1004763

**Published:** 2023-05-04

**Authors:** Brice Passera, Sylvain Harquel, Alan Chauvin, Pauline Gérard, Lisa Lai, Elena Moro, Sara Meoni, Valerie Fraix, Olivier David, Estelle Raffin

**Affiliations:** ^1^CNRS UMR 5105, Laboratoire Psychologie et Neurocognition, LPNC, Grenoble, France; ^2^Univ. Grenoble Alpes, Inserm, U1216, CHU Grenoble Alpes, Grenoble Institut Neurosciences, Grenoble, France; ^3^Berenson-Allen Center for Noninvasive Brain Stimulation, Division of Cognitive Neurology, Department of Neurology, Beth Israel Deaconess Medical Center and Harvard Medical School, Boston, MA, United States; ^4^CNRS, INSERM, IRMaGe, Grenoble, France; ^5^Defitech Chair in Clinical Neuroengineering, Neuro-X Institute and Brain Mind Institute, EPFL, Geneva, Switzerland; ^6^Aix Marseille Univ, Inserm, U1106, INS, Institut de Neurosciences des Systèmes, Marseille, France

**Keywords:** Parkinson’s disease, deep brain stimulation, transcranial magnetic stimulation, TMS-EMG mapping, TMS-EEG mapping, state-dependent mapping, DBS-TMS paired-pulse mapping

## Abstract

**Introduction:**

Transcranial magnetic stimulation (TMS) mapping has become a critical tool for exploratory studies of the human corticomotor (M1) organization. Here, we propose to gather existing cutting-edge TMS-EMG and TMS-EEG approaches into a combined multi-dimensional TMS mapping that considers local and whole-brain excitability changes as well as state and time-specific changes in cortical activity. We applied this multi-dimensional TMS mapping approach to patients with Parkinson’s disease (PD) with Deep brain stimulation (DBS) of the sub-thalamic nucleus (STN) ON and OFF. Our goal was to identifying one or several TMS mapping-derived markers that could provide unprecedent new insights onto the mechanisms of DBS in movement disorders.

**Methods:**

Six PD patients (1 female, mean age: 62.5 yo [59–65]) implanted with DBS-STN for 1 year, underwent a robotized sulcus-shaped TMS motor mapping to measure changes in muscle-specific corticomotor representations and a movement initiation task to probe state-dependent modulations of corticospinal excitability in the ON (using clinically relevant DBS parameters) and OFF DBS states. Cortical excitability and evoked dynamics of three cortical areas involved in the neural control of voluntary movements (M1, pre-supplementary motor area – preSMA and inferior frontal gyrus – IFG) were then mapped using TMS-EEG coupling in the ON and OFF state. Lastly, we investigated the timing and nature of the STN-to-M1 inputs using a paired pulse DBS-TMS-EEG protocol.

**Results:**

In our sample of patients, DBS appeared to induce fast within-area somatotopic re-arrangements of motor finger representations in M1, as revealed by mediolateral shifts of corticomuscle representations. STN-DBS improved reaction times while up-regulating corticospinal excitability, especially during endogenous motor preparation. Evoked dynamics revealed marked increases in inhibitory circuits in the IFG and M1 with DBS ON. Finally, inhibitory conditioning effects of STN single pulses on corticomotor activity were found at timings relevant for the activation of inhibitory GABAergic receptors (4 and 20 ms).

**Conclusion:**

Taken together, these results suggest a predominant role of some markers in explaining beneficial DBS effects, such as a context-dependent modulation of corticospinal excitability and the recruitment of distinct inhibitory circuits, involving long-range projections from higher level motor centers and local GABAergic neuronal populations. These combined measures might help to identify discriminative features of DBS mechanisms towards deep clinical phenotyping of DBS effects in Parkinson’s Disease and in other pathological conditions.

## 1. Introduction

With the development of increasingly focal stimulation techniques, better spatial targeting with neuronavigation and realistic head models, transcranial magnetic stimulation (TMS) has become a useful tool for exploratory studies of the organization of the human corticomotor representation, particularly under conditions of altered physiology such as motor neuron diseases (Chase, 2014), stroke (Smith and Stinear, 2016), Parkinson’s disease (Cantello et al., 2002) or to study plasticity of the corticospinal tract after specific interventions (e.g., Raffin and Siebner, 2019; Gaffney et al., 2021). When TMS is applied to the primary motor cortex (M1), it activates the corticospinal neurons and generates motor evoked potential (MEP) in the target muscles. Classical motor mapping with TMS offers a non-invasive probe of motor cortical representation in humans. It can evaluate features of motor representations and be used to draw conclusions about muscle group somatotopy and plasticity within M1 (Pascual-Leone et al., 1994; Kleim et al., 2007). For TMS-based corticomotor mapping, a focal figure-of-eight shaped coil is discharged over a grid of scalp positions and the amplitude of the Motor Evoked Potentials (MEPs) are recorded from a contralateral target muscle for each grid site, enabling the construction of a corticomotor map for the target muscle (Wilson et al., 1993). This muscle-specific corticomotor map is thought to contain spatial information about its functional cortical representation in the precentral motor cortex.

Later developments in the field further improved spatial resolution and reliability of TMS mapping outcomes, by adjusting or individualizing coil orientations (Bashir et al., 2013; Raffin et al., 2015), stimulation grids using subject-specific gyrification (Raffin et al., 2015; Nazarova et al., 2021; Numssen et al., 2021), by using multimodal information derived from corticospinal tract tractography (Weiss Lucas et al., 2017; Muir et al., 2022), by automating motor stimulation protocols (Harquel et al., 2017) or by taking into account cortical projections (Bungert et al., 2017). These developments led to high-resolution (<10 mm2) individual mapping of several hand muscle representations in M1. Other propositions were made to improve feasibility especially by decreasing the time needed to complete a motor mapping session, a crucial aspect in clinical settings (van de Ruit et al., 2015).

When using single-pulse TMS, the temporal resolution of TMS can also be very high and can provide information about brain functions on the order of milliseconds (de Graaf et al., 2014; Ficarella and Battelli, 2019). As a result, TMS can also map state dependent brain dynamics using task-based modulation of excitability (Cattaneo and Silvanto, 2008; Duecker et al., 2013). In this situation, TMS is used as a gateway for accessing or altering neural dynamics during a specific time window of a behavior. The possibility to use temporal TMS mapping to trace state-dependent shifts in excitability profiles allows to define causal models of the relationships between specific activated or inhibited neural regions and functional changes in behavior. Still in the temporal domain, it is possible to pair a preceding conditioning pulse applied to M1 or to a remote cortical area/ sub-cortical area with a test pulse applied to M1 to probe intracortical circuitry in the motor cortex (Ilić et al., 2002) or in the parietal cortex for instance (Oliveri et al., 2000) but also effective connectivity from one remote area to M1 (Koch and Rothwell, 2009; van Campen et al., 2013).

Another recent development consists in mapping whole brain reactivity by coupling TMS with electroencephalography (TMS-EEG; Rogasch and Fitzgerald, 2013; Harquel et al., 2016a). TMS paired with EEG offers an opportunity to examine cortical reactivity of “silent” brain regions, i.e., regions that do not produce direct or measurable TMS outcome (Tremblay et al., 2019). Our group already applied this approach to healthy participants, to demonstrate different intrinsic neurodynamical properties in different stimulated regions (Harquel et al., 2016a; Raffin et al., 2020) even to dissociate neurodynamical activity within the sensorimotor area (Passera et al., 2022). This TMS-EEG coupling approach can therefore provide useful insights on whole-brain effects on an intervention with a ms scale temporal resolution.

All in all, TMS mapping can be multi-dimensional (spatial, temporal) and multi-scale (from within-hand motor mapping to whole brain mapping). Bringing all these approaches together can provide the unique opportunity to draw a full neurophysiological picture on a specific research question. In this study, we leveraged all these proxies for local/whole-brain excitability and measures of temporal neural dynamics under various cognitive/physiological states, into a multi-dimensional and multi-scale TMS mapping approach. To illustrate the unique potential of this framework, we applied it to patients with Parkinson’s disease (PD) treated with Deep Brain Stimulation of the Subthalamic Nucleus (DBS-STN; Groiss et al., 2009) to illustrate how this approach can provide complementary information DBS mechanisms.

PD is a neurodegenerative condition affecting approximatively 1% of individuals over 60 (Tysnes and Storstein, 2017) originating from the degeneration of midbrain dopaminergic neurons and neuronal alpha-synuclein inclusions (Kalia and Lang, 2015; Surmeier et al., 2017). Akinesia, bradykinesia, rigidity and resting tremor are key clinical hallmarks of the disease (Kalia and Lang, 2015), together with a plethora of non-motor symptoms that dramatically impair quality of life (Schapira et al., 2017). Regarding akinesia, evidence have shown that PD patients respond more quickly or easily when their actions are in response to environmental stimuli (i.e., exogenously evoked) than when they are self-initiated (endogenously evoked; Brown and Marsden, 1988; Hallett, 2008). Despite the widely accepted clinical evidence, the deficit in endogenous movement initiation and programming has been sporadically quantified in patients (Cohen et al., 2015) and the effects of DBS-STN on the endogenous-exogenous asymmetry when initiating a movement, are unknown. The therapeutic principle of DBS for PD is that high frequency DBS-STN regulates the dysfunctional output from local neural circuits. Clinically efficient protocols are thought to result in a dissociation of input and output signals in the basal ganglia, resulting in the disruption of abnormal information and a loss of output specificity in a broader motor network (Chiken and Nambu, 2014; Hamani et al., 2017; Schor et al., 2022).

TMS can be safely applied in movement disorders patients with DBS implanted in different targets in the basal ganglia (Udupa and Chen, 2015; Udupa et al., 2016; Phielipp et al., 2017) and has already disclosed multiple functional alterations of the corticospinal pathway in PD (Cantello et al., 2002; Underwood and Parr-Brownlie, 2021). Studying M1 physiology is of special interest in PD because midbrain dopaminergic neurons influence the firing rate and synchronization of M1 neurons (Parr-Brownlie and Hyland, 2005; Vitrac et al., 2014; Grandi et al., 2018) through direct projections and indirect pathways involving the basal ganglia and motor thalamus (Galvan et al., 2015). The clinical relevance of M1 activity in the physiopathology of PD is further demonstrated by the studies showing improvements in motor symptoms following repetitive transcranial magnetic stimulation (TMS) of M1 (Khedr et al., 2003; Yang et al., 2018; Yokoe et al., 2018). Furthermore, M1 activity appears to be influenced by the established PD therapies such as L-DOPA (Fierro et al., 2008; Monte-Silva et al., 2010) or DBS-STN (Fraix et al., 2008; Dejean et al., 2009; Kuriakose et al., 2010; Devergnas and Wichmann, 2011; Johnson et al., 2020). But these later studies investigating M1 changes following DBS-STN did not concurrently explore the multiple facets of corticomotor changes that may occur differently in patients. Furthermore, it is likely that the DBS effects are not limited to the motor cortical neurons but they might spread out to the broader (motor) networks (Shang et al., 2020). They might even manifest differently in other neuronal groups, especially those areas that are in charge of the neural control of movements [i.e., the inferior frontal gyrus (IFG) and the pre supplementary motor area (preSMA)] and that are also the main targets of the integrative activity of basal ganglia.

Finally, for a full exploration of DBS-STN effects on M1 activity, it is possible to use single-pulse DBS combined with single pulse TMS triggered on the induced cortical evoked potentials recorded by electroencephalography and measure how motor cortical excitability is directly modulated by STN inputs on a single-trial basis (Kuriakose et al., 2010; Udupa and Chen, 2015; Ni et al., 2019). This measure could represent a complementary indicator of antidromic activation of the corticosubthalamic hyperdirect pathway, as one of the possible target of DBS-STN.

In this study, we used multi-dimensional and multi-scale TMS mapping to provide a proof-of-principle that this approach can map a given motor symptoms’ profile in patients to specific electrophysiological or behavioral profiles, mediating beneficial DBS outcomes.

DBS exerts its effects through multiple circuits that might differently impact behavior, corticospinal excitability and whole brain dynamics. We hypothesized that high frequency DBS will regulate the control of action, in particular by normalizing endogenous-exogenous asymmetry when needing to initiate movements. Additionally, we expected acute corticomotor reorganization and changes in neural dynamics among the key motor areas in proportion with symptoms improvements. We also expected that single STN inputs would dynamically modulate corticospinal excitability in a time dependent manner. Altogether, this proof-of-concept study aims to illustrate the exciting potential of this multi-scale, multi-dimensional TMS mapping approach, as it opens new avenues towards TMS mapping based phenotyping, potentially transposable to multiple clinical conditions.

## 2. Methods

### 2.1. Patients

This study was conducted at the IRMaGe TMS facility in collaboration with the Movement disorders Neurology department of Grenoble Alpes University Hospital. Six patients (1 women) took part in the study (noted PKM01 to PKM06). They all signed a written consent following a thorough description of the study by a neurologist. All patients were diagnosed with Parkinson’s Disease (PD) for at least 9 years (maximum: 15 years). They all experienced motor fluctuations before bilateral subthalamic nucleus deep brain stimulation implantation. They were operated at least 12 months before inclusion. Demographics and clinical features are summarized in Table 1. The disease profile of the patients was homogenous since all were mostly akinetic and rigid (see Table 2 for the sub-scores of the MSD-UPDR scale).

**Table 1:** Patient description with age (years), gender (M: male, F: female), laterality (R: right-handed, L: left-handed), Time since diagnostic (years), clinical parameters for STN-DBS, type of dual channel neurostimulators and MMSE (Mini-mental score examination).

#	Age	Gender	Laterality	Time since diagnosis	DBS clinical parameters	DBS stimulators	MMSE (/30)
PKM01	68	M	R	10	R: 2.4 V L: 2.2 V	ACTIVA PC (Medtronic ®)	30
PKM02	64	F	R	15	R: 1.5 mA L: 1.2 mA	VERCISE GEVIA (Boston Sci ®)	28
PKM03	59	M	L	10	R: 2 mA L: 3.3 mA	VERCISE GEVIA (Boston Sci ®)	30
PKM04	62	M	R	10	R: 2.5 V L: 2.3 V	ACTIVA PC (Medtronic ®)	28
PKM05	63	M	R	11	R: 1.6 V; 3.3 V	ACTIVA PC (Medtronic ®)	27
PKM06	69	M	R	9	R: 2.0 V; L: 2.5 V	ACTIVA PC (Medtronic ®)	29

**Table 2:** MDS-UPDRS score ON and OFF STN-DBS and sub-scores for akinesia, tremor and gait/balance.

#	DBS-OFF	Total MDS-UPDRS (/132)	Akinesia subscore (/40)	Tremor subscore (/32)	Gait and balance subscore (/20)	DBS-ON	Total MDS-UPDRS (/132)	Akinesia subscore (/40)	Tremor subscore (/32)	Gait and balance subscore (/20)
PKM01		55	22	3	10		24	9	0	8
PKM02		42	22	0	6		29	16	0	7
PKM03		40	20	0	6		19	12	0	1
PKM04		29	13	0	7		20	8	0	4
PKM05		33	16	0	6		16	7	0	2
PKM06		37	20	0	4		18	9	0	3

Inclusion criteria comprised no contraindication to TMS, no psychiatric or neurologic pathologies outside of PD, normal cognitive function (MMSE ≥ 24) and they had to be equipped with MRI compatible Deep Brain Stimulation (neurostimulators, see Table 1). Patients were recruited for this study as part of their one-year follow-up visit at the Neurology department. To capture only DBS related effects, all dopaminergic medication related to PD treatment was stopped at least the night before each session. All procedures used were approved by an ethical committee (ID/RCB: 2017-A03016-47) and respected the Helsinki declaration for safety.

### 2.2. Study design

**Figure 1:**
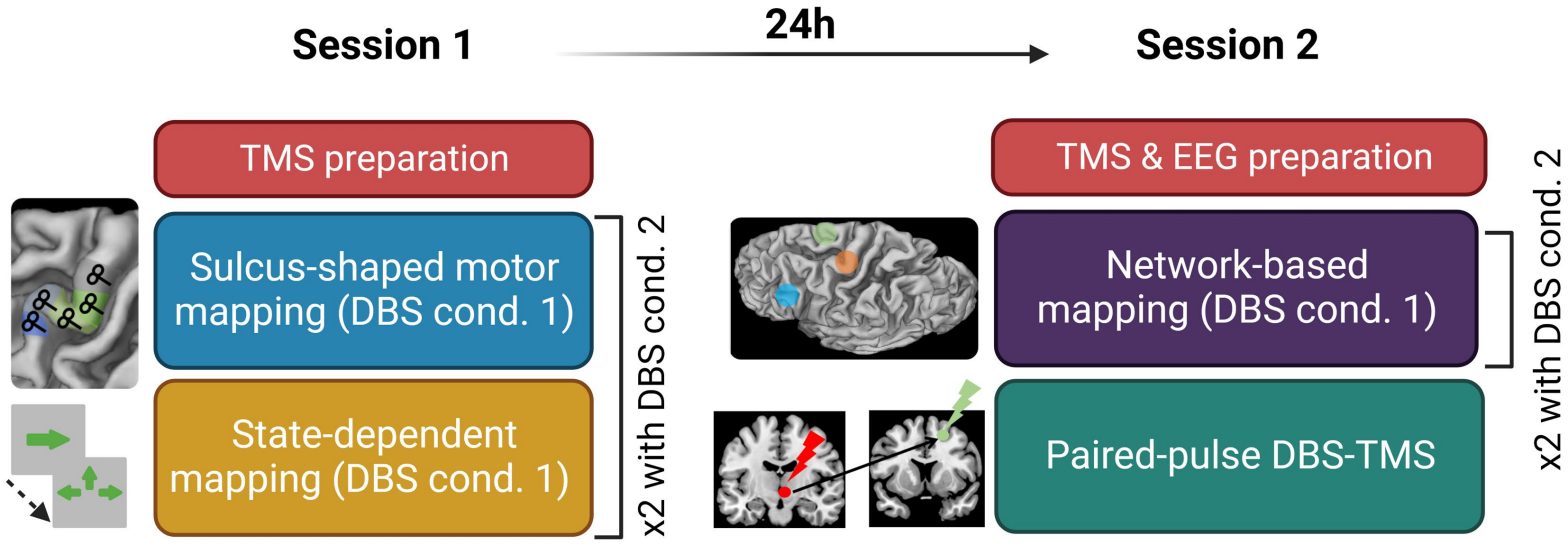
Experimental design detailing the content of Session 1 and Session 2; cond., condition; TMS, transcranial magnetic stimulation; DBS, deep brain stimulation; EEG, electroencephalography.

All patients performed two sessions and each session took place early in the morning to minimize the duration of the off-medication state (Figure 1). Each session was composed by two similar experimental blocks, the STN-DBS stimulation being randomized and consecutively ON and OFF (or vice versa). The study was double blinded, a clinical research assistant anonymized the order of the STN-DBS stimulations conditions (ON/OFF). A neurologist or nurse, not involved in the experiment, came between conditions to change the stimulation parameters. A T1-weighed MRI acquired pre-surgery was used for the neuronavigation system. For each participant, the MRI was processed in the neuronavigation software before the first visit. The targeted hemisphere was defined as the hemisphere where the patient presented the most severe motor symptoms (with no tremor).

### 2.3. Sulcus-shaped TMS motor mapping procedures

The first session started with the hotspot hunting procedure. An exploratory grid of 5×5 targets spaced by 7 mm centered on the anatomical hotspot was used. Once the experimenter found the hotspot, defined as the point eliciting the most reliable and the highest MEP, the resting motor threshold (rMT) was assessed using TMSMTAT (Awiszus, 2011). The first experimental stimulation condition was then set (DBS ON or OFF) and the TMS-EMG motor mapping began. The sulcus-shaped mapping approach of the precentral gyrus consisted in five targets placed along the bending of the right central sulcus and centered around the handknob, with a coil orientation of 45° with respect to the wall of the central sulcus. Targets were spaced by at least 1 cm from one another. The order of target stimulation was varied across subjects but maintained constant within patients, in the ON and OFF states Each of the five targets was stimulated with 20 single biphasic TMS pulses at an intensity of 120% RMT and an inter-stimulus interval (ISI) of 3 to 5 s. This sulcus-shape based, linear TMS mapping method yields a one-dimensional spatial representation of the corticomuscular excitability profile in M1 (Raffin et al., 2015; Dubbioso et al., 2017; Madsen et al., 2019; Raffin and Siebner, 2019; Figure 1B).

### 2.4. Network-based TMS-EEG mapping procedures

This exam aimed at mapping the cortical excitability within the movement initiation network with STN-DBS ON and OFF. For both sessions, targets were defined using projection of cortical targets derived from MNI coordinates {for the supplementary motor area (SMA [±6 8 72])} and the inferior frontal gyrus (IFG [±60 24 13]), using SPM12 normalization for the patient’s anatomy. The session started by the EEG cap mounting and rMT assessment over the motor hotspot previously defined. The first DBS stimulation condition was set at the end of the parametrization phase. Each session consisted in two experimental blocks consisting in a TMS-EEG mapping (in both DBS ON and OFF condition). TMS-EEG mapping consisted in the stimulation of three cortical areas involved in motor control: M1, the supplementary motor area (SMA) and the inferior frontal gyrus (IFG). A hundred biphasic pulses were delivered on each target at 0.5–0.7 Hz with a stimulation intensity of 120% of rMT, corrected for scalp-to-cortex distance according to the Stokes formula using the following formula:


$$\mathrm{AdjMT}\%=\mathrm{MT}+3\times \left(D_{\mathrm{siteX}}-D_{\mathrm{M1}}\right)$$

where AdjMT% is the adjusted motor threshold (MT) in percentage stimulator output, MT is the unadjusted MT in percentage stimulator output, D_M1_ is the distance between the scalp and M1, D_SiteX_ is the distance between the scalp and a second cortical region (SiteX), and 3 is the spatial gradient relating MT to distance, estimated at 3 (Stokes et al., 2005, 2007). Current direction was perpendicular the cortical sulcus underneath the TMS coil to maximize and homogenize the neuronal activation. A realistic sham condition was also performed in each block (Raffin et al., 2020). Precisely, the TMS coil was flipped on the placebo side, no magnetic pulse was delivered, but an electrical stimulation was delivered concurrently to each TMS pulse through two skin electrodes (stimulating area of 10 × 6 mm^2^) placed in a bipolar montage near electrodes AF4 and F6. The electrical stimulation intensity was tuned individually for each subject, in order to mimic muscular twitches or skin sensations comparable in terms of strength, pain, or discomfort, to active TMS pulses.

### 2.5. State-dependent TMS mapping procedures

To explore whether STN-DBS modifies dynamic changes in corticospinal excitability during movement initiation, all patients performed a task-TMS paradigm that measured shifts in MEP size during the motor preparation phase and their reaction times (RT; pushing a joystick) following self-selected action preparation (endogenous cues) versus exogenously presented cues. The experimental task was coded and run on Matlab using the Psychophysics Toolbox (Kleiner et al., 2007; ref). Patients remained seated in the chair of the robotic TMS while staring at the presentation screen centered at eye level and placed at a comfortable viewing distance between 60 and 100 cm. After the presentation of a 1,000 ms fixation cross, they were asked to move the joystick as fast and accurately as possible towards the green arrow displayed during the 500 ms cue. They held the joystick with their most impaired hand (see 2.2) and had up to 1,500 ms to respond. “Exogenous” cues consisted in one green arrow pointing towards one direction. “Endogenous” cues consisted in multiple choices among three of the four cardinal directions. Individual reaction times, based on the joystick movement latency, were extracted from a baseline block (without TMS) of 50 trials for both Endogenous and Exogenous conditions prior the experiment, after 40 training trials. The onset of the biphasic single pulse TMS was individually set, 150 ms before movement onset, with an intensity of 120% rMT. Patients were told to perform the task as fast and as accurate as possible and to avoid systematic bias in one direction for the Endogenous condition. To exclude any specific effect of TMS over M1 on reaction times, TMS was applied only on half of the trials. We recorded a total of 50 trials for each condition (“Endogenous-TMS,” “Exogenous-TMS,” “Endogenous-noTMS,” “Exogenous-noTMS”). Note that in the DBS-OFF, PKM01 and PKM03 did not entirely complete the task because of symptoms worsening. However, more than the first half of the trials were available and kept for analysis.

**Figure 2:**
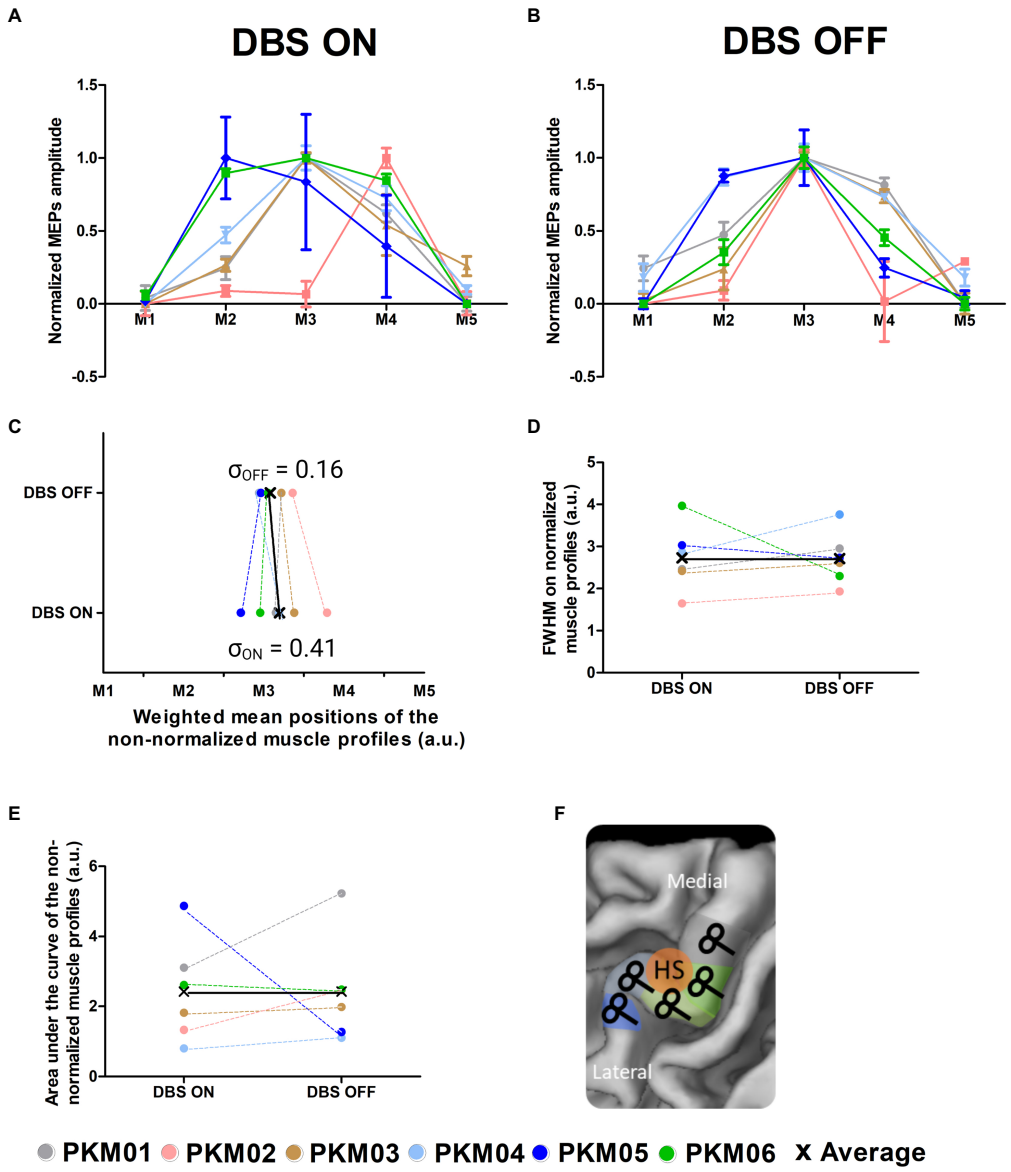
**(A)** Corticomotor excitability profiles of the 6 patients obtained with DBS ON; **(B)** Corticomotor excitability profiles of the 6 patients obtained with DBS OFF; **(C)** Global excitability indexed by the area under the curve computed from the non-normalized corticomotor excitability profiles in the ON and OFF states; **(D)** Focality of the excitability profiles measured as the full width at half maximum computed from the normalized corticomotor excitability profiles in the ON and OFF states; **(E)** Weighted Mean Position of the excitability profiles in the ON and OFF states; **(F)** Illustration of the TMS motor mapping where TMS sites are individually defined along the central sulcus (M1 to M5); HS, hotspot.

### 2.6. Paired-pulse TMS mapping procedures

For each participant, we also performed an experimental block of 300 pulses on M1. This block consisted of biphasic paired pulse stimulation between the STN-DBS and M1. We tested two ISI of 4 and 20 ms, together with a third ISI of 180 ms that serves as a control condition, based on result found by Udupa and Chen (2015). For this procedure, the ISI were randomized, and the stimulations were delivered by block of 100 pulses. Patients’ stimulators frequencies were set at 3 Hz, the minimal frequency for the neurostimulators. To trigger TMS pulses, we used surface electrodes placed on participant’s neck over the wires connecting the stimulator to the stimulation electrodes. The EMG software was set to trigger a TMS pulse once it detected a DBS pulse, with a minimum ISI of 3 s. The inter-trial jitter of the delay between DBS and TMS stayed below 1 ms, since no jittering was noticeable *a posteriori* between the two artifacts during EEG processing.

### 2.7. Data acquisition

#### 2.7.1. TMS

Biphasic TMS pulses were delivered using a B65-RO A/P, double sided coil (Magventure, Denmark) plugged into a Magpro x100 TMS stimulators (Magventure, Denmark). The coil was positioned perpendicularly to the gyrus and was robotically handled (Axilum Robotics, France) and neuronavigated (Localite, Germany) during all TMS sessions.

#### 2.7.2. EMG

EMG data were recorded using a Cambridge Electronic device system (CED, Cambridge, GB), sampled at 5 kHz, and processed using Signal (CED, Cambridge, GB). Electrodes were placed on the hand presenting the least number of tremors in a tendon-belly montage for the first dorsal interosseus with the ground electrode placed on the ulna. More relevant to the behavioural task, EMG electrodes were also placed over two other extrinsinc hand muscles, the agonist flexor (FCR) and antagonist extensor (ECR) carpi radialis.

#### 2.7.3. EEG

EEG data was recorded using a 128-channels active cap and TMS compatible system (BrainAmp DC amplifiers, and ActiCap, Brain products, GmbH, Germany). At the start of Session #2, the cap was placed according to the 10–20 standard system. Impedance levels were adjusted and kept under 5 kOhms using conduction gel. Impedance was checked between each block and adjusted if necessary. EEG signal was recorded with the amplifier in DC mode with an anti-aliasing filter and digitized at 5 kHz sampling frequency. The reference and ground electrodes were Fz and AFz, respectively. To reduce the impact of the TMS click, patients were equipped with noise cancelling earbuds (Bose QC-25). A small layer of plastic was placed on the coil’s surface to reduce any sensory impact. At the end of session two, electrodes positions were recorded using the neuronavigation software.

### 2.8. Data processing

#### 2.8.1. Behavioral analyses

Reaction times were extracted for the two different conditions (“Exogenous cues” and “Endogenous cues”) and the two DBS conditions (“ON” and “OFF”). Additionally, we compared for the Endogenous condition, the distribution of the movement directions (left, right, up and down) normalized by the total number of cues presentation for each direction. This will allow us to capture changes in motor repertoire or in cognitive flexibility with DBS ON and OFF.

#### 2.8.2. EMG processing

The EMG signals were processed using CortexTool (Harquel et al., 2016b), a Matlab toolbox developed in the lab and freely available online. EMG data were band-pass filtered (50–600 Hz), any trials presenting muscle activity in the baseline were removed. MEPs were visually inspected and automatically detected. The peak-to-peak amplitude of MEPs was extracted using Signal software in the time window between 10 and 40 ms after the TMS stimulus (Cambridge Electronic Design, Cambridge, UK). For the first dorsal interossei (FDI) muscle, we constructed mediolateral corticomotor excitability profiles based on the mean MEP amplitudes for each TMS target site along the central sulcus forming the hand knob. We derived three complementary measures from the MEP amplitude profiles to study in more detail dynamic changes in the muscle-specific representations in M1 with or without DBS. The area under the curve (AUC) was taken as an index sensitive to a global up or downscaling in corticomotor excitability and computed using the *trapz* function in Matlab. The “amplitude-weighted mean position” (WMP) of the FDI excitability profiles was used to assess changes in topographical representation of the FDI. The amplitude-WMP was calculated according to the following formula:


$$WMP=\frac{\sum_{k=1}^{7}\;Target\;\left(k\right)*Mean\;MEP\;AmplitudeTarget\;\left(k\right)}{\sum_{k=1}^{7}\;Mean\;MEP\;AmplitudeTarget\;\left(k\right)}$$

Finally, for display purpose and to appreciate the shape of the curves, we normalized each corticomotor excitability profiles using this formula:


$$norm\;excit.profile=\;\;\frac{\begin{array}{l} (Mean\;MEP\;AmplitudeTarget\;\left(k\right)\\ -\min.\left(MEP\;AmplitudeTarget\;\left(1:5\right)\right) \end{array}}{\begin{array}{l} \max.\left(MEP\;amplitudeTarget\;\left(1:5\right)\right)\\ -\min.\left(MEP\;AmplitudeTarget\;\left(1:5\right)\right) \end{array}}$$

Using these normalized excitability profiles, we computed the Full Width at Half Maximum (FWHM) to reflect the sharpness of the FDI muscle representation using the customized Matlab scripts.

#### 2.8.3. EEG preprocessing

EEG signal was semi-automatically processed using Fieldtrip (Oostenveld et al., 2011) with home-made script written in Matlab (The MathWorks Inc., United States), using (Rogasch et al., 2014) two-rounds independent component analysis (ICA) method. First, a visual inspection of each trial and each channel was performed to remove channels with electrical noise (flat signal or amplitude >100 μV). Then, the signal was epoched from −1,000 to +1,000 ms around the TMS pulse. The signal was then cut from −5 to +15 ms to remove the TMS stimulation artifact. For the ON condition, an additional signal processing step was applied to accurately cut the signal in consideration with the DBS induced artifacts (see Supplementary Figure S1A ). The method was based on the derivative of the absolute raw signal. For each trial, the two edges of the time window were adjusted so that they fall within a period of at least 10 ms of flat signal (blue arrows in Supplementary Figure S1A). This allowed to avoid strong signal amplitude discontinuities between the two edges that would have randomly appeared otherwise from one trial to another, and that would have induced strong interpolation artifacts in the following preprocessing steps (see the blue dotted line in comparison of the red dotted line in Supplementary Figure S1A). A first round of independent component analysis was then executed to identify and remove the muscle artifact. Trials were then interpolated using spline interpolation and auto-regressive models, band-pass filtered (1–80 Hz) and re-reference using average reference. A second step of visual inspection was performed to remove bad trials for each condition and a second round of ICA was then executed. Noisy components (i.e., blinks, decay artifacts, auditory-evoked potentials, muscle contractions and other noise-related artifacts) were visually identified using time-series and topography and then removed. Notably, remaining DBS artifacts were efficiently highlighted during this step (see Results). Clean EEG times-series were then reconstructed on rejected channel using the average activity of neighboring channels. Finally, the EEG activity was symmetrically flipped relative to the central axis for patients who were stimulated on the right hemisphere, so that all the stimulation targets were on the left.

Note that the double ICA preprocessing framework effectively removed DBS-induced artifacts in the ON condition (Supplementary Figure S1A). Depending on the patient and site, between 5 and 15 components (over 110+) were affected by this artifact and thus removed from the signal. They were easily spotted on the basis of their time courses and their topographies showed stereotyped patterns, with either activation on the outer perimeter (“crown” like activation, see Supplementary Figure S1A) or highly localized activation around the crossing of the stimulator wires behind the ears (unilateral temporo-parietal activation, see for example Supplementary Figure S1A). The efficiency of the procedure, i.e., the absence of any residual DBS artifact, was visually checked on both single trials and evoked potentials.

#### 2.8.4. TMS-evoked potentials and local mean field potential

TEP were computed for each target, stimulation condition and patient by averaging the EEG signal across trials using baseline normalization (−500 to −50 ms, Z-score). Local Mean Field Power (LMFP, μV2) was calculated using the non z-scored TEP on the 6 to 7 electrodes closest to each target, and to Cz for sham conditions (M1: [‘FCC3h’ ‘C1’ ‘C3’ ‘CCP3h’ ‘CP1’ ‘CP3’]; SMA: [‘FCC1h’ ‘FC1’ ‘FFC1h’ ‘F1’ ‘FFC3h’ ‘FCC3h’]; IFG: [‘F5’ ‘FC5’ ‘FFT7h’ ‘F7’ ‘FFT9h’ ‘FT7’]; Sham: [‘C1’ ‘Cz’ ‘C2’ ‘CCP1h’ ‘CCP2h’ ‘FCC1h’ ‘FCC2h’]). Finally, LMFP amplitude of ISI 4 and ISI 20 conditions from the paired-pulse TMS mapping were normalized in respect with their control condition ISI 180, prior to the statistical analysis.

#### 2.8.5. Statistics

Descriptive statistics were performed on the behavioral and EMG data using GraphPad Prism software version 8.0 for Windows (GraphPad Software, San Diego, CA, USA). Additionally, a Wilcoxon signed-rank test was performed on the EMG derived measures and a Friedman test was applied on reaction times with factors *DBS state* (ON vs. OFF) and *Movement type* (Endogenous vs. Exogenous) as it accounts for non-gaussian distribution and for relatively low-sample sizes. The variability in the muscle representation between ON and OFF states was assessed by calculating the standard deviation of the weighted mean positions along central sulcus at the group level. The difference between ON and OFF states was then statistically assessed using a permutation test, where the observed “true” difference between the two standard deviations was compared with those obtained from the 32 possible permutations, the final *p*-value being the proportion of permutations resulting to a stronger difference than the observed one.

Differences in the amplitude of LMFP extracted from TMS-EEG recordings were analyzed using repetitive measure ANOVA (rmANOVA), with *stimulation site* (M1, DLPFC, SMA) and *DBS state* (ON vs. OFF) as factors. Pairwise comparisons between LMFP amplitudes of DBS ON vs. OFF within a similar stimulation site, and between ISI 4 and ISI 20 for the paired-pulse mapping, were investigated using Wilcoxon signed-rank tests. For all the above-mentioned tests, the significance level was set to α = 0.05.

## 3. Results

All patients tolerated well the off-medication state. Note that one patient had freezing of gait in OFF MED-ON DBS condition (PKM02) and another one had unilateral upper limb rest tremor in OFF MED and OFF DBS condition (PKM01).

None of the patients reported adverse effects with respect to the TMS-DBS coupling. Clinical scores reflected the worsening of the motor state in the OFF DBS state with a mean total motor MDS-UPDRS (part III) score of 35/132 (SD = 6) in the OFF DBS state and 23.20/132 (SD = 4.9) in the ON state (see Table 2 of individual data). As expected, sub-scores revealed marked improvements in the akinesia sub-scores [18.8 (3.6)/40 in the OFF DBS state and 10.2 (3.3)/40 in the ON DBS state].

### 3.1. Sulcus-shaped TMS motor mapping

Sulcus shape-based TMS mapping was used to map the corticomotor representations of the FDI muscle of the less affected side in each individual (see Figure 2F). Sulcus shape-based mapping showed that DBS triggered a reorganization of the FDI representation, which involved changes in corticomotor excitability and spatial representation (Figures 2A,B). Corticospinal excitability was measured as AUC, representing the mean MEP amplitude for all five-map positions. No significant change in global excitability was reported at the group level (W = −7, *p* = 0.56). Note that two patients showed marked changes, an increase in excitability with DBS ON for PKM05 and a decrease in excitability for PKM01. The sharpness of the muscle profiles was evaluated using the FWHM, which showed a trend for a decrease in focality at the group-level, although non-significant (W = −5, *p* = 0.34). Interestingly, while there was no difference in the actual mean position of the muscle representation along the central sulcus in the ON state compared to the OFF state (W = −3, *p* = 0.84), there was a significant larger inter-individual variability in the weighted mean positions (permutation test, *p* = 0.03). The dispersion of the FDI representation in the ON state was nearly four times higher than the one observed in the OFF state, as reflected by the standard deviation of the weighted mean positions along the central sulcus (σ_ON_ = 0.41, σ_OFF_ = 0.16, see Figure 2C). This reflects slightly more erratic arrangement of the FDI representation along the hand knob area of M1. In contrast, as also visible in the normalized excitability profiles in Figure 2B, DBS OFF was associated with a clustering of the FDI representation around target 3 (M3), which identifies the exact center of the hand knob area even when considering the actual position of the curve’s peaks.

### 3.2. State-dependent TMS mapping

**Figure 3:**
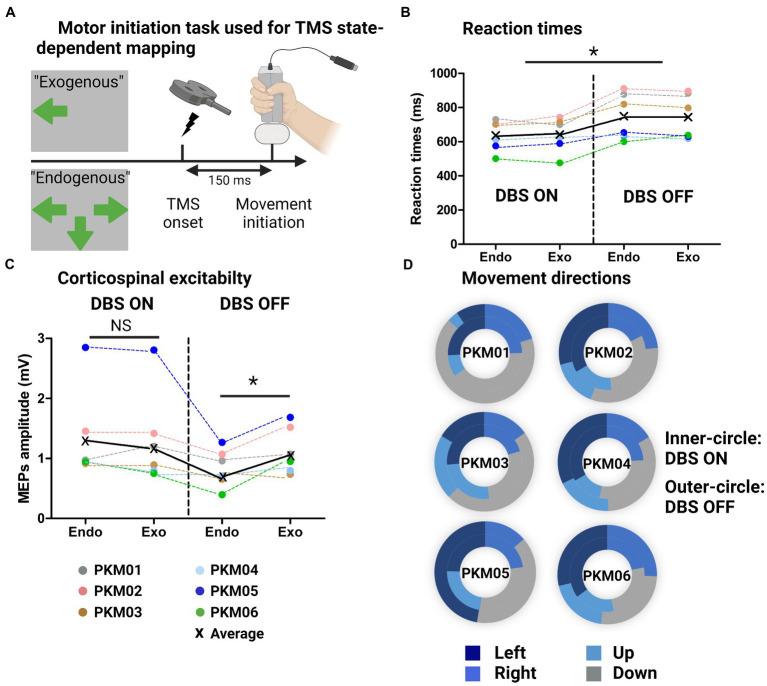
**(A)** Illustration of the behavioral task with the Exogenous condition (externally triggered) and endogenous condition (self-initiated from multiple responses choice), showing the TMS onset triggered 150 ms before movement initiation; **(B)** Individual and group average reaction times at the behavioral task for the Endogenous and Exogenous cues in the ON and OFF DBS states. **(C)** Individual and group average MEPs amplitudes in the same four conditions; **(D)** Doughnut plots showing in the inner-circle the number of occurrences of up/down/left/right choices (Endogenous condition) in the ON state and in the outer-circle, in the OFF state.

To assess state dependent modulations of corticospinal excitability, we used a movement initiation task (Cohen et al., 2015), which randomly alternates between endogenous (multiple choices of self-initiated actions) and exogenous cues (externally triggered). Single-pulse TMS was applied over M1 in approximately half of the trials during the movement preparation phase (i.e., 150 ms before the actual start of the movement) (see Figure 3A). All patients were able to perform the task in the ON and OFF DBS state (except PKM01 and PKM03 who had to stop half-way through the task because of symptoms worsening). Accuracy was stable across conditions except for PKM03 who showed a massive drop in accuracy in the OFF DBS state, below chance level (see Table 3).

**Figure 4:**
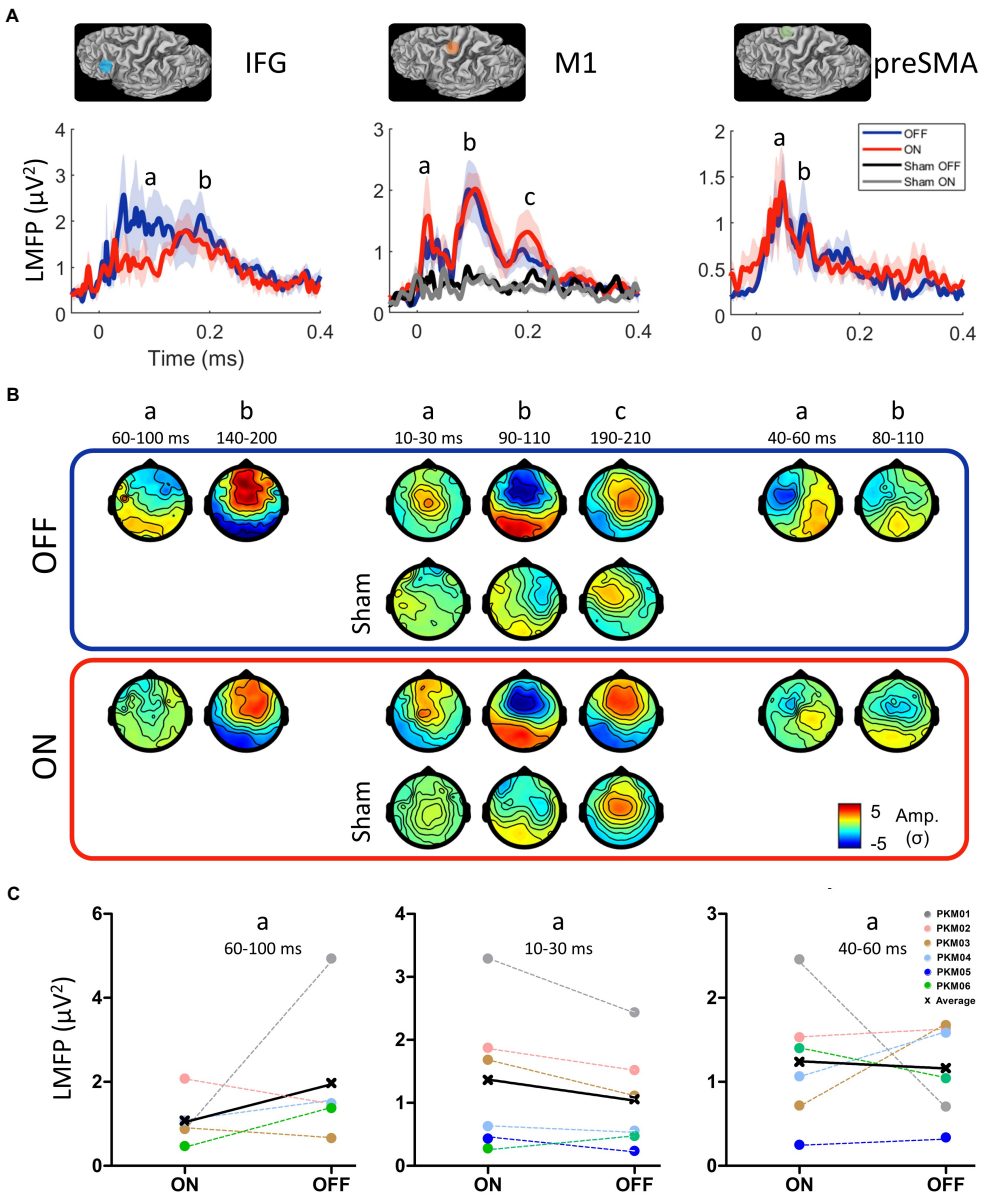
**(A)** Location of the three stimulation sites belonging to the motor initiation network (IFG: inferior frontal gyrus, M1: primary motor cortex, preSMA: pre-supplementary motor area) and the grand average of their respective local mean field power (LMFP) in the ON (red) and OFF (blue) states and the Sham condition in the ON and OFF states. Shaded areas indicate the standard error of the mean. **(B)** Grand average TEP topographies (Z-score) of the local EEG activity for the three sites at the significant time points extracted from the LMFP in the ON and OFF states. **(C)** Mean LMFP of the earliest activity peak from the three sites, in the ON and OFF states.

**Table 3:** Individual and group average in task accuracy for the 8 experimental conditions.

Accuracy (%)	DBS ON				DBS OFF			
	TMS		No TMS		TMS		No TMS	
Patients	Exogenous	Endogenous	Exogenous	Endogenous	Exogenous	Endogenous	Exogenous	Endogenous
PKM01	92	88	96	94	90	68	89	71
PKM02	98	100	100	100	100	100	100	100
PKM03	98	94	100	96	37	33	40	15
PKM04	92	100	80	96	94	100	84	96
PKM05	64	79	67	72	64	79	72	72
PKM06	100	98	100	94	100	95	94	100
Average	**91**	**89**	**90**	**88**	**80**	**82**	**81**	**80**

We first examined the differences in reaction times (RT) with respect to DBS state and motor cues (Endogenous versus Exogenous) without TMS. Figure 3 shows improved reaction times when DBS was ON as revealed by a significant main effect of DBS (*χ^2^* = 8.1, W = 0.7, *p* = 0.005). However, we found no effect of movement type (*χ^2^* = 0, W = 0, *p* = 1). The MEP amplitudes associated with these four conditions are displayed on Figure 3B. Although there was no main effect of DBS (*χ^2^* = 2.4, W = 0.25, *p* = 0.12) nor Movement type (*χ^2^* = 1.1, W = 0.03, *p* = 0.30). The interaction between those two factors was significant and revealed a significant drop in corticospinal excitability for Endogenous movements in the OFF state (W = 21, p = 0.03). This electrophysiological marker was specific to Endogenous movements as there was no difference in MEP amplitudes for Exogenous movements (W = 13, *p* = 0.7). Finally, we extracted a proxy for executive dysfunctions by looking at the lack of flexibility in motor control. To do so, we compared the occurrence of joystick movements in the four directions with DBS ON and OFF (normalized by the maximal number of possible occurrences for each direction). To quantify some potential bias towards the same direction, we compared the standard deviation (SD) in movement directions for all patients in the ON and OFF state (0 reflecting no systematic bias). Although the group average was not different (W = 6, *p* = 0.44), the SD in the ON state was 10.49 [range: 5.2–14.6] while it was largely increased in the OFF state (16.26 [6.7–33.4]). Note that some patients (in particular PKM01, PKM03, PKM05) did show a strong bias towards always the same motor response where they were offered multiple choices, denoting a decrease in motor flexibility when DBS was OFF (Figure 3C).

### 3.3. Network-based TMS-EEG mapping

All patients underwent the TMS-EEG mapping procedure at all three cortical sites without any adverse effects. However, the IFG condition (both ON and OFF) was not acquired in PKM05 due to excessive fatigue. The grand average of the LMFP after DBS artifact removal (see Supplementary Figure S1) over the three regions of interest (IFG, pre-SMA and M1) and realistic sham condition are displayed on Figure 4A, while TEP topographies are displayed on Figure 4B. Overall, all the active TMS conditions evoked early and late activity that could be strongly differentiated from the baseline (from 3 to 6 σ higher than baseline), whereas the realistic sham conditions evoked late and weaker activity starting from 100 ms (between 2 and 3 σ). The rmANOVA conducted on the early part of LMFPs showed a tendency for an effect of site [*F*(3,4) = 4.2, *p* = 0.06], and no effect for DBS condition [*F*(1,4) = 0.58] nor interaction [F(3,4) = 0.56]. Qualitatively, the activity evoked by the IFG stimulation showed the strongest difference between the ON and OFF DBS conditions. The early signal on the LMFP was higher in the OFF DBS condition over the 60 to 100 ms time-period, before getting closer to the LMFP of the ON DBS condition after 140 ms. This difference was not significant at the group level (W = 11, p = 0.44), being mainly driven by PKM01 and PKM06, with 2 patients showing opposite effect direction (Figure 4C). Over M1, the early component was stronger in the ON compared to the OFF condition from 10 to 30 ms, both in terms of peak amplitude and spatial spread of the evoked activity. Although not reaching the significance level at the group level (W = 2, *p* = 0.09), this effect was consistent with 5 over 6 patients showing this pattern (Figure 4C). Finally, there was no evidence for a significant modulation of activity evoked by pre-SMA TMS stimulation by DBS ON compared to DBS OFF (W = 12, *p* = 0.84), the inter-patient variability regarding the effect direction and size of DBS conditions being higher in this site (Figure 4C).

### 3.4. Paired-pulse TMS mapping

**Figure 5:**
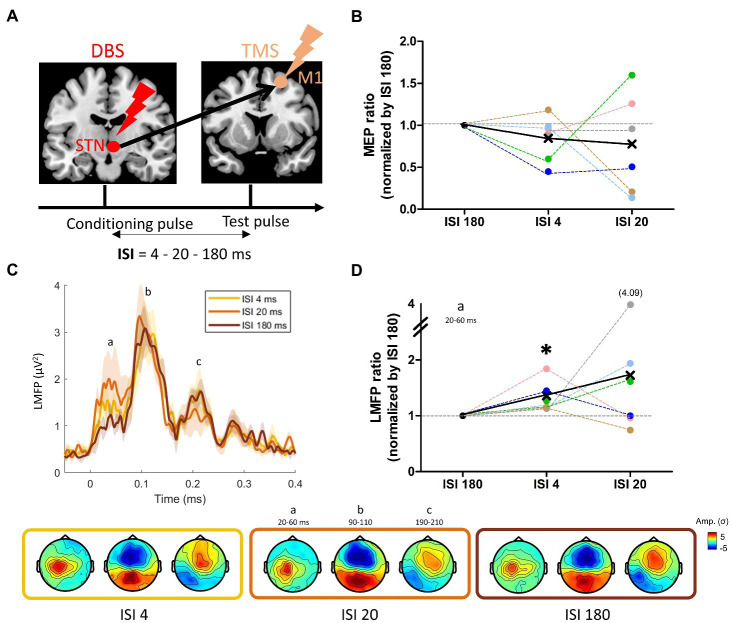
**(A)** Paired pulse DBS-TMS principle, with a DBS-STN single pulse followed by a TMS pulse over M1 at three different intervals: 4, 20, and 180 ms; **(B)** Changes in motor evoked potentials (MEP) induced by the STN condition pulse at the three ISIs. **(C)** Grand average of the LMFP induced by the paired-pulse DBS-TMS procedure at the three ISIs. Shaded areas indicate standard error of the mean. Topographies induced by the three ISI at the time points defined with the LMFP (a: N45-P30, b: N100, c: P200) are displayed on the bottom row, from ISI 4 to ISI 180 (left to right). **(D)** Mean LMFP of the earliest activity peak for the three ISI, normalized (ratio) by ISI 180. The black star indicates a significant difference from 1 (see text).

The paired-pulse DBS-TMS paradigm is illustrated in Figure 5A, in which three different ISI were tested: 4, 20, and 180 ms. Visual inspection of Figure 5B suggests that the conditioning effect of the single DBS-STN pulse on M1 corticospinal excitability was on average inhibitory, especially for ISI 4 were 5 out of 6 patients presented this inhibition, although not significant at the group level (ISI 4: W = −18, *p* = 0.16; ISI 20: W = −12, *p* = 0.38). Of note, the individual data revealed a large inter-individual variability in the DBS-STN conditioning effect, as shown for instance by the strong facilitatory effect of patient PKM06 and PKM02 at ISI 20. At the cortical level, while the TMS-evoked electric fields remained similar in terms of spatial distribution (Figure 5C), the LMFP showed amplitude modulations across the three ISIs (Figure 5D). Notably, these modulations were concentrated on the early part of the response between 20 and 60 ms, where both the 4 and 20 ms ISIs led to an increase in the local signal amplitude. At the group level, the increase of early local activity was significant for ISI 4 (W = 21, *p* = 0.03), with an average increase of 0.5 μV^2^ (+36%) compared to ISI 180. Despite the fact that this effect appeared stronger for ISI 20 (with an average increase of 1 μV^2^ corresponding to +73%), it was not significant at the group level (W = 16, *p* = 0.31).

### 3.5. An integrative perspective

**Figure 6:**
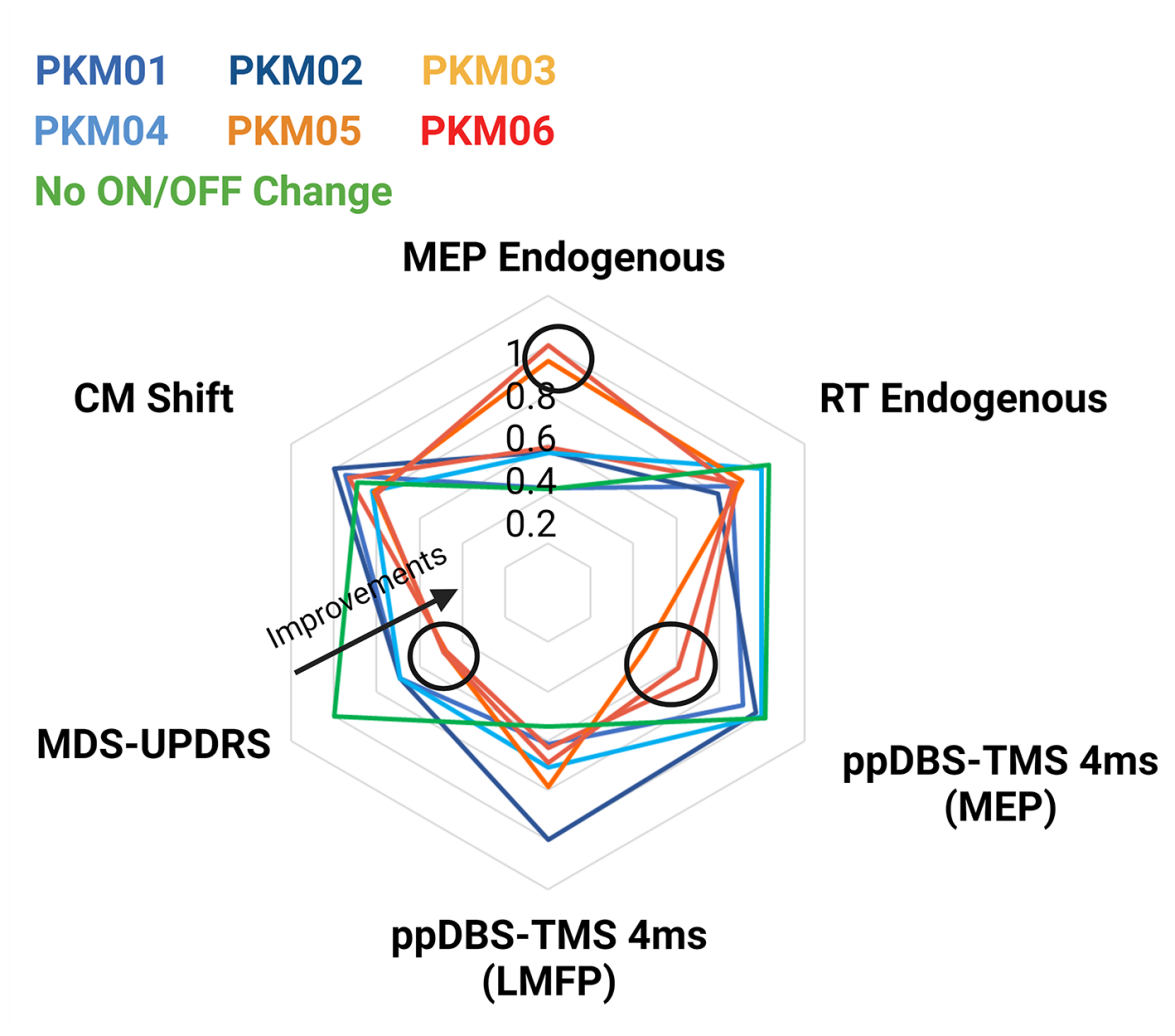
Radar plot showing individual MDS-UPDRS scores and the corresponding variables that showed significant group effects (reaction times, MEPs, and ppDBS-TMS). Patients in hot colors were the ones demonstrating larger motor improvements.

We plotted the individual changes in the MDS-UPDRS score when DBS was ON and OFF along with the variables that showed significant ON/OFF group differences (i.e., the reaction times and the MEPs associated with Endogenous movements, the paired pulse DBS-TMS measured with LMFP - and the associated MEPs - with an ISI of 4 ms and the shift in finger muscle’s representations in M1). This figure aimed at investigating whether the variability within the DBS effects can predict greater symptoms improvements. The radar plots showed that the patients who were the most improved with DBS ON (PKM03, 05 and 06) had larger decreases in reaction times and increase in MEP amplitude when initiating an endogenous movement. These patients also significantly more inhibition when M1 was conditioned by a single DBS pulse over the STN (Figure 6). Those patients were also the ones who improved the most on the akinesia sub-score (see Table 2).

## 4. Discussion

Rather than looking unidimensional on the relationship between cortical excitability and DBS, the complementary set of TMS mapping approaches presented here highlights some new potential mechanisms of action underlying DBS effects.

The primary motor cortex plays a key role in motor control and is one of the primary output of the motor portions of the basal ganglia (Alexander et al., 1986). Therefore, previous studies have compared motor cortical excitability and cortical circuits in patients with movement disorders with DBS ON and OFF to elucidate the underlying mechanisms of DBS in Parkinson’s disease (Lozano et al., 2002) but have yielded to mixed results. Some studies revealed a reduction of cortical excitability measured with MEP size produced by TMS over M1 in the ON state (Cunic et al., 2002; Wessel et al., 2016), but only in moderate Parkinson’s disease patients. DBS STN seems not to normalize the increased MEP in advanced Parkinson’s disease patients (Chen et al., 2001; Cunic et al., 2002; Wessel et al., 2016). These studies also report a normalization of intracortical circuits measured with paired-pulse TMS protocols over M1 (Cunic et al., 2002; Fraix et al., 2008). Earlier PET imaging studies revealed a decrease in regional cerebral blood flow in M1 at rest, which might reflect the reduced activity of excitatory neurons induced by STN stimulation (Limousin et al., 1997). Of importance, findings from other DBS applications suggested the existence of differential effects depending on TMS intensity. For instance, increased MEP amplitude was reported with high but not with low TMS intensity (Kühn et al., 2003). From another perspective, Weaver and colleagues showed that DBS reduces the current intensity necessary to modulate motor-evoked potentials induced by focally applied direct cortical stimulation (Weaver et al., 2020), suggesting in contrast an increased excitability with DBS ON. This increase in excitability is somehow supported by our EMG data (Figure 2C), together with our TMS-EEG data, which tended to show a modulation of M1 activity within the 10 to 30 ms time-window when DBS was ON (Figure 4), such component (P30) being positively linked with the excitability of the corticospinal system (Darmani and Ziemann, 2019; Passera et al., 2022).,

In this study, we used a sulcus-based motor mapping approach and showed at the EMG level, rather than a global and systematic change in direct corticomotor excitability, a qualitative change in motor outputs selectivity. DBS produced an acute reorganization of corticomotor neurons, possibly reflecting a loss of sub-cortical-to-cortical output specification through the direct and indirect pathways, possibly explaining the mixed results in the literature. While the core representation of the FDI muscle clustered around one similar central target in the OFF state, we measured a large variability across patients in the ON state, with FDI representations peaking more medially and for others more laterally. A somewhat similar observation has been previously reported in nonhuman primate model of PD, i.e., a very large variability in antidromic activation induced by DBS-STN (Johnson et al., 2020). In our data, it can be speculated that the inter-individual differences in DBS lead placement partly explain the variability in topological location of M1 cells that are antidromically modulated by DBS. Some medial M1 axons could be preferentially activated in some patients and more lateral M1 axons for others, supporting a medial or lateral shift of corticospinal excitability along M1. Alternatively, these mediolateral shifts might be explained by different distributions of intracortical neurons. There is evidence from human studies that DBS of the STN has a direct effect on intracortical neurons, modifying the balance between excitation and inhibition (Fraix et al., 2008), resulting in an acute remodeling of corticospinal projections. Potential candidates underlying this fast cortical remodeling are the local inhibitory and excitatory interneurons in M1 (Siebner et al., 2022).

In many clinical contexts, some pathophysiological components become even more explicit or are over-expressed during active brain states or even during specific functional tasks (Gilio, 2003; Boyadjian et al., 2011). In the present study, we measured MEPs in the preparatory phase of endogenous (self-initiated) and exogenous (externally triggered) movements of extrinsic hand muscles involved in the task. Impaired endogenous action is largely related to an enhanced sensory guidance in Parkinson’s patients (Torres et al., 2011). The impaired context-dependent action initiation is not only a pure motor phenomenon, but appears to involve a complex interplay between motor, cognitive, and affective components (Heremans et al., 2013), including action selection and initiation (Tard et al., 2014), dual-tasking (Spildooren et al., 2010), and the ability to switch cognitive and motor sets (i.e., executive functions; Amboni et al., 2008), providing a global index of cognitive motor control in PD. Past PET studies and event-related cortical potential studies showed altered cortical activity prior to self-initiated actions (Papa et al., 1991; Jahanshahi et al., 1995). At the behavioral level, we report some improvements in reaction times with DBS-ON compared to DBS-OFF. Although it is a commonly reported result in the literature (Werheid et al., 2007; Cohen et al., 2015), our study might have been underpowered to reveal a statistical difference between the endogenous and exogenous conditions. Importantly, corticospinal excitability showed a marked drop in the preparatory phase of the endogenous condition in the DBS OFF state. While there is evidence that the basal ganglia-thalamo-motor loop has been shown to be impaired during self-triggered actions (Papa et al., 1991; Taniwaki et al., 2013), to the best of our knowledge, it is the first demonstration of a pathological modulation of corticospinal excitability, specific to self-triggered actions in PD. This impaired corticospinal excitability might reflect a lack of drive to initiate endogenous movements. Additionally, motor flexibility was very perturbed for some patients who tended to systematically choose the same movement direction despite multiple choices, in the DBS OFF state. We propose that this automatization of motor control or lack of motor repertoire could reflect the fact that the dopaminergic projection to the STN provides a signal for implicit “motor motivation,” similar to the established role of dopamine in explicit reward-seeking behavior (Bromberg-Martin et al., 2010). Thus, without these inputs, patients show a resistance to plan “new” motor commands because of the energy expenditure required. Furthermore, our results showed that DBS restored the level of corticospinal excitability in the endogenous condition in all but one patient, and improved reaction times when self-initiated actions were involved. Interestingly, the patients who benefit the most from DBS showed higher amount of corticomotor reorganization.

Complementarily, we investigated cortical excitability within the movement control network composed here by the preSMA, the IFG and M1 monohemisperically. Our results showed a decrease in IFG excitability when STN-DBS was ON. Considering the inhibitory role of IFG in motor control (Kenner et al., 2010; Rae et al., 2015; Aron et al., 2016; Chen et al., 2020), it is possible that the abnormal IFG over-excitability found in the OFF mode reflects excessive inhibitory inputs coming from the IFG to M1. STN-DBS has been shown to trigger GABA modulation in the basal ganglia and thalamus (Stefani et al., 2011; Buchanan et al., 2014; Alosaimi et al., 2022). Our results suggest that clinically efficient STN-DBS might also partially exert its beneficial effects through a transient GABAergic disinhibition propagating to the cortex. This abnormal inhibitory inputs might impair movement initiation, and movement withholding (Zhang et al., 2017). Similar to IFG, the preSMA neurons appear to be involved in action control, in particular in inhibitory motor control (Swann et al., 2012; Zhang and Iwaki, 2019). However, our results did not show any modulation of the preSMA using single pulse TMS in the ON and OFF DBS state. A recent meta-analysis specifically reviewed neuroimaging data in relationship with akinesia and found little, if no evidence for any change in resting-state preSMA/SMA activity in patients with PD showing akinesia in comparison to task-based studies (Spay et al., 2019). This is congruent with our results since the dominant symptom in our sample of patients was akinesia. Then, the modulation of the preSMA by STN-DBS could indeed be state-dependent and arise only during motor preparation or inhibition (Berardelli et al., 2001; Blasi et al., 2006). In order to test this, delivering preSMA TMS pulses during a motor initiation task could reveal cortical excitability modulation induced by STN-DBS (Chen et al., 2020). In particular in the context of the motor task used in the present study, previous studies have suggested that the deficit in self-initiated movements in Parkinson’s disease is due to supplementary motor area underactivation (e.g., Jahanshahi et al., 1995).

Altogether, the TMS-EEG data revealed local disinhibitory effects of DBS over M1, and a reduction of exaggerated inhibitory inputs from the IFG. However, while pharmacology of the primary motor cortex has been extensively studied, the equivalent in the frontal cortex needs to be addressed in future research. These modulations of inhibitory signals might be mediated by different intracortical circuitries, directly or indirectly supporting distinct clinical effects. Our present sample size was too small to run sub-group analyses, but one can speculate that the down-regulation of IFG inputs to M1 supports improvements of akinesia symptoms. In contrast, the normalization of GABA-mediated inhibition within M1 might be beneficial for tremor. M1 has been closely related to tremor generation (Leodori et al., 2022) and is partly vanished under dopaminergic drugs, interacting with GABAergic agents (Shukla et al., 1988).

STN-DBS has probably many different effects on neurons in the stimulated STN and *via* the cortico-basal ganglia loop through orthodromic activation of efferent axons, antidromic and orthodrimic activation of afferent axons. The antidromic activation is visible using cortically evoked potentials, using sclap-EEG recordings (e.g., Ashby et al., 2001; Udupa and Chen, 2015). Ashy and colleagues demonstrated a first negative peak of evoked potential at a short latency (2–8 ms) and later studies have reported a positive peak of evoked potential around 18–25 ms (MacKinnon et al., 2005; Kuriakose et al., 2010). The nature of the connectivity between STN and M1 can be further explored by investigating the effect of a single-pulse DBS on MEP amplitudes using a single-pulse TMS over M1. For instance, Hanajima et al., showed an early facilitation of M1 (approximately 3 ms after the STN pulse; Hanajima et al., 2004) and later Kuriakose et al., showed the same phenomenon using later latencies (18 to 25 ms after the STN pulse; Kuriakose et al., 2010). The first facilitatory peak is thought to originate from an antidromic activation of the hyperdirect pathway from the cortex to STN while the later peak might be mediated by synaptic activation through the indirect pathway through the motor thalamus. We tested these two intervals (4 and 20 ms) compared to a longer interval (180 ms) where no long-lasting STN modulations on M1 were expected. The MEP results showed a slight inhibitory effect at the group level but interestingly the patients who were the most improved by DBS were the ones with the strongest inhibitory effect (Figure 6). These differences might reflect different involvements of the direct and hyperdirect pathways (Dunovan et al., 2015). The TMS-EEG data also revealed consistent modulations of EEG activity with the largest effect produced by the longest interval between DBS and TMS (20 ms). EEG signals from 20 to 60 ms were particularly modulated (Figure 5). This interval is centered on the N45, a component that has been associated with inhibitory neurotransmission through GABA-A receptors (Premoli et al., 2014). As a result, it appears that STN acts on M1 at these precise latencies by boosting inhibitory circuits (Chen et al., 2020). Larger studies, potentially involving multimodal neuroimaging and multi-centric patients’ recruitment will be needed to unravel which of the pathways connecting STN, not only to M1 but to other parts of the brain, are the most relevant for DBS actions (Chen et al., 2020).

## 5. Conclusion

This proof-of-principle study using multi-dimensional and multi-scale TMS mapping approach revealed that DBS acts on multiple components of the motor system. Besides providing new knowledge on cortical remapping occurring within the primary motor cortex and in secondary motor centers, our results showed that the largest clinical benefits of DBS were associated with a normalization of context-dependent modulation of corticospinal excitability and stronger recruitments of inhibitory circuits. This important finding might indicate potential clinical features exploitable for deep phenotyping of clinical DBS effects. This technic might then represent a new opportunity to safely scan DBS effects in a lot of clinical contexts, not only in the motor domain, provide objective and complementary data, increase our knowledge of its mechanism, enhance evaluation and fine tuning of DBS parameters, and ultimately, improve patient care.

## Data availability statement

The raw data supporting the conclusions of this article will be made available by the authors, without undue reservation.

## Ethics statement

The studies involving human participants were reviewed and approved by ID/RCB: 2017-A03016-47. The patients/participants provided their written informed consent to participate in this study.

## Author contributions

BP and SH designed the experiments, collected the data, performed the analyses, and wrote the paper. AC performed the analyses and reviewed the paper. PG and LL collected the data and performed the analyses. SM and EM helped with patient’s recruitment, data collection, and reviewed the paper. VF designed the experiments, recruited the patients, collected the data, provided fundings, and reviewed the paper. OD designed the experiments, provided fundings, and reviewed the paper. ER conceived and designed the experiments, performed the analyses, wrote the paper, and provided fundings. All authors contributed to the article and approved the submitted version.

## Funding

This work was funded by the Agence Nationale pour la Recherche grant “ANR-15-CE37-0015-1” and by NeuroCoG IDEX UGA in the framework of the “Investissements d’avenir” program (ANR-15-IDEX-02). Data were acquired on a platform of France Life Imaging Network partly funded by the grant “ANR-11-INBS-0006.”

## Conflict of interest

The authors declare that the research was conducted in the absence of any commercial or financial relationships that could be construed as a potential conflict of interest.

## Publisher’s note

All claims expressed in this article are solely those of the authors and do not necessarily represent those of their affiliated organizations, or those of the publisher, the editors and the reviewers. Any product that may be evaluated in this article, or claim that may be made by its manufacturer, is not guaranteed or endorsed by the publisher.

## Supplementary material

SUPPLEMENTARY FIGURE S1 **(A)** Illustration of the DBS-induced artifact removal on the EEG raw data. Red and blue arrows indicate the intended and corrected time window for cutting the TMS artifact out. Red and blue dotted lines depict the resulting discontinuity between edges and its correction; **(B)** Two representative examples of ICA components driven by the DBS artifact.
