# Supplementary figures and images for: Multi-scale and cross-dimensional TMS mapping: A proof of principle in patients with Parkinson’s disease and deep brain stimulation
Source: Front Neurosci. 2023 May 4;17:1004763. doi: 10.3389/fnins.2023.1004763 (PMC10192635; doi:10.3389/fnins.2023.1004763)

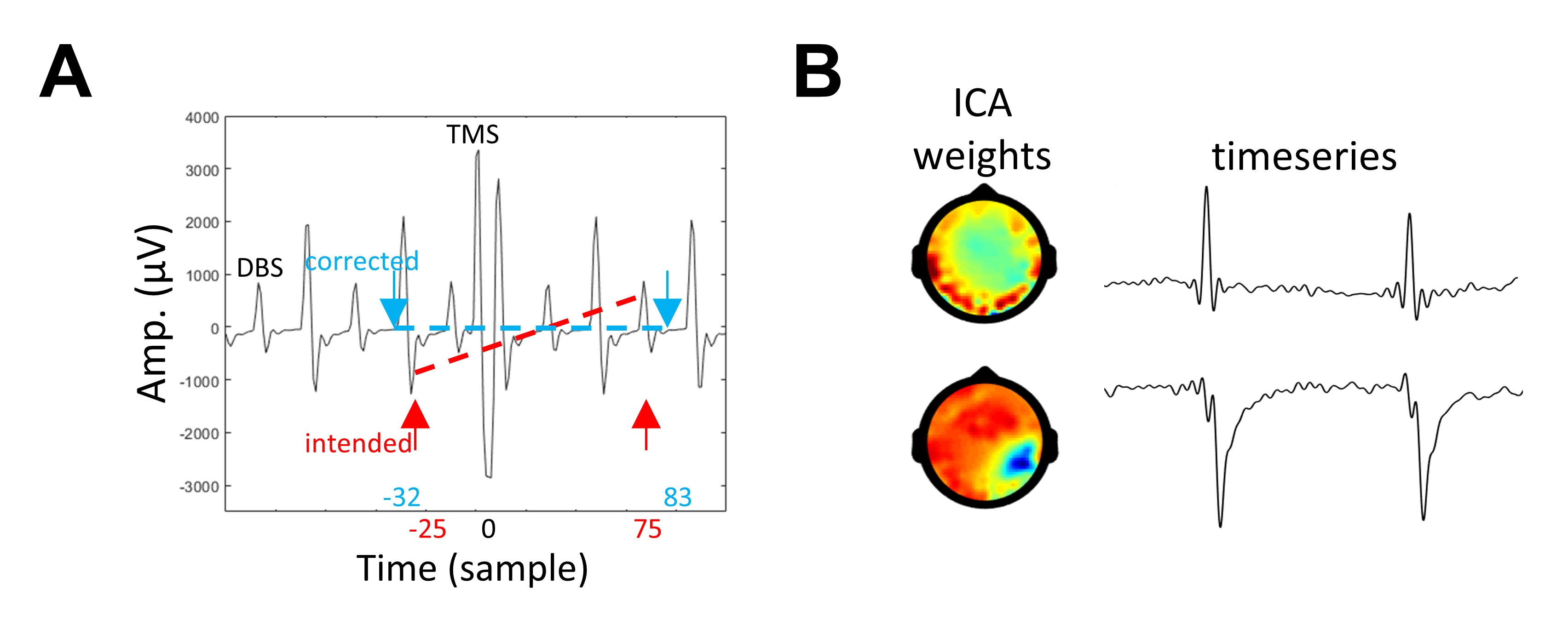

Supplement: SUPPLEMENTARY FIGURE S1 — (A) Illustration of the DBS-induced artifact removal on the EEG raw data. Red and blue arrows indicate the intended and corrected time window for cutting the TMS artifact out. Red and blue dotted lines depict the resulting discontinuity between edges and its correction; (B) Two representative examples of ICA components driven by the DBS artifact. [file Image_1.jpg]
